# The effect of obstructed action efficacy on reward-based decision-making in healthy adolescents: a novel functional MRI task to assay frustration

**DOI:** 10.3758/s13415-021-00975-w

**Published:** 2021-12-29

**Authors:** Katia M. Harlé, Tiffany C. Ho, Colm G. Connolly, Alan N. Simmons, Tony T. Yang

**Affiliations:** 1 VA San Diego Healthcare System, San Diego, CA; 2 Department of Psychiatry, University of California San Diego, La Jolla, CA; 3 Department of Psychiatry and Behavioral Sciences, Division of Child and Adolescent Psychiatry, Weill Institute for Neurosciences, University of California San Francisco, San Francisco, CA; 4 Department of Biomedical Sciences, Florida State University, Tallahassee, FL

**Keywords:** frustration, fMRI, reward processing, action efficacy, adolescents

## Abstract

Frustration is common in adolescence and often interferes with executive functioning, particularly reward-based decision-making. Yet, very little is known about how incidental frustrating events (independent of taskbased feedback) disrupt the neural circuitry of reward processing in this important age group.

While undergoing functional magnetic resonance imaging (fMRI), 45 healthy adolescents played a card game in which they had to guess between two options to earn points, in low- and high-stake conditions. Functioning of button presses through which they made decisions was intermittently blocked, thereby increasing frustration potential.

Neural deactivation of the precuneus, a Default Mode Network region, was observed during obstructed action blocks across stake conditions, but less so on high-relative to low-stake trials. Moreover, less deactivation in goal-directed reward processing regions (i.e., caudate), frontoparietal ‘task control’ regions, and interoceptive processing regions (i.e., somatosensory cortex, thalamus) were observed on high-stake relative to low-stake trials.

These findings are consistent with less disruption of goal-directed reward seeking during blocked action efficacy in high-stake conditions among healthy adolescents. These results provide a roadmap of neural systems critical to the processing of frustrating events during reward-based decision-making in youths and could help characterize how frustration regulation is altered in a range of pediatric psychopathologies.

## 1. Introduction

The experience of frustration, i.e., a state associated with being blocked from attaining one’s goal (Dollard et al., 1939; Berkowitz, 1989), is common in adolescence (Deveney et al., 2013; Perlman et al., 2015; Tseng et al., 2019). Frustration can lead to increased irritability which is a key clinical symptom of several forms of important psychopathology and behavioral problems, including depressive disorders, anxiety disorders, substance use disorders, bipolar disorder, disruptive mood dysregulation disorder, oppositional defiant disorder, intermittent explosive disorder, and conduct disorder (Bava and Tapert, 2010; Stringaris et al., 2013; Jeronimus et al., 2017). Repeatedly experiencing frustrating events may have a significant impact on emotion regulation and mental health in adolescents, as it disrupts goal-based behavior, including reward-based decision-making (Brotman et al., 2017). Deficits in reward-based decision-making have, in turn, a tangible impact on the obtainment and pursuit of rewards such as social contacts (Davey et al., 2008), furthering the experience of missed opportunities and worsening in irritability and depressive symptoms (Forbes et al., 2009). This interplay between frustration, depressive symptoms, and reward sensitivity is therefore highly relevant to adolescent health.

While reward processing has been investigated in relation to frustration among youth, this prior work has focused on the impact of irritability, defined as a tendency to experience tonic frustration (Adleman et al., 2011; Kryza-Lacombe et al., 2021), or the broader profile of adolescent depression (Forbes et al., 2006; Silk et al., 2012) on reward processing. Few studies have investigated the impact of discrete frustrating events during reward-based decision-making in youth. In this work, frustration has been equated to omission of rewards (Perlman et al., 2015; Brotman et al., 2017), or created by providing “rigged” task feedback (Rich et al., 2011; Deveney et al., 2013). However, within a goal-directed framework of decision-making, which assumes individuals anticipate and strategize to obtain rewards, these conceptualizations of frustration may introduce significant confounds. Specifically, omitting rewards or providing negative feedback in an otherwise normal context are likely to modify individuals’ beliefs about the reward environment (action-outcome contingencies) and the usefulness of their strategy. This may, in turn, bias their decision-making as individuals attempt to address the discrepancy between altered expectations and observed outcomes (e.g., biased reward prediction errors).

An alternative way to conceptualize frustrated reward processing is through incidental (i.e., not directly related to the observation and learning of action-reward contingencies) perturbations to goal-directed decision-making. Such manipulations are more likely to interrupt rather than change one’s goal-directed strategy or perception of reward availability. For instance, individuals’ sense of agency and perceived controllability of the environment determine their ability to observe and predict reward outcomes, which, in turn, impacts their goal-directed behaviors and emotional responses to the environment (Moscarello and Hartley, 2017; Dorfman and Gershman, 2019). Perception of low controllability during goal-directed tasks is obstructive to goal attainment, which is associated with negative affect, primarily frustration and anger (Van Steenburg et al., 2013; Maglio et al., 2014). Thus, modulating the level of perceived control and agency in the context of goal-directed reward-seeking, for instance by obstructing one’s ability to observe action-outcome contingencies, may help shed light on important neurocognitive mechanisms underlying frustration-related behavior in adolescents.

To address this goal, we developed a novel experimental paradigm which is based upon a reward-based decision-making fMRI task paradigm previously validated in adult (Delgado et al., 2005) and adolescent populations (Forbes et al., 2006; Forbes et al., 2009). With the goal of maximizing reward, this novel task introduces a manipulation aimed at decreasing perceived action efficacy (i.e., low controllability). Here, we define “action efficacy” as the experience that a taken action has the desired effect. Obstructed action efficacy is operationalized as being prevented from implementing goal-directed actions through intermittent blocking of the effects of button presses, a process which is independent of reward outcomes and task difficulty. The primary goal of this study was to test the usefulness of this novel paradigm in characterizing frustrated reward processing in adolescents. To that end, we first aimed to characterize the neural circuity involved in the experience of obstructed reward-based decision-making (i.e., low agency context) in a healthy adolescent sample. Given the importance of reward magnitude to reward anticipation processes (Delgado et al., 2000; Knutson et al., 2001) and observed reward processing abnormalities in adolescents (Bjork et al., 2004), we examined how the degree of motivation, as captured by stake condition (i.e., low vs. high potential gains), may further modulate the neural response to obstructed action efficacy.

Goal-directed reward-based decision-making is supported by a frontostriatal circuit (Bjork et al., 2004; Delgado et al., 2005; Hare et al., 2008), which overlaps with the central executive network, including the dorsomedial and dorsolateral prefrontal cortex and posterior parietal areas. Obstructions to controllability or agency are also likely to prompt neural changes in the default mode network (DMN), a set of brain regions characterized by reduced neural activity during cognitive tasks, which includes the ventromedial prefrontal cortex and the posterior cingulate cortex/precuneus regions (Raichle et al., 2001; Fair et al., 2008). Thus, we hypothesized that obstruction of action efficacy when making reward-based decisions would be associated with a) reduced neural activity in areas involved in goal-directed reward seeking, particularly the frontoparietal/central executive network (e.g., dorsolateral and dorsomedial PFC, posterior parietal regions) and the ventral striatum; and b) deactivations of DMN regions. This pattern would reflect perseveration and maintenance of task engagement despite reduced reward processing. Action efficacy obstruction during high-stakes relative to low-stake condition should be associated with higher frustration potential as action blocking occurs in the context of greater motivation to obtain reward. Moreover, while the DMN network is reliably deactivated during engagement in a cognitive task (Raichle et al., 2001), weaker disengagement of DMN regions and recruitment of salience regions (including amygdala and anterior insula) and somatosensory areas (including sensorimotor areas and posterior insula) are observed in response to frustrating and other emotionally salient events in adults (Abler et al., 2005; Pawliczek et al., 2013; Yu et al., 2014; Satpute and Lindquist, 2019), with emerging evidence of similar patterns in youths (Perlman et al., 2015; Li et al., 2017; Tseng et al., 2019). Therefore, we hypothesized that being blocked on high-stake relative to low-stake trials would result in higher affective and interoceptive processing, reflected by less deactivation of the DMN and stronger recruitment of regions within affective salience and somatosensory/ interoceptive processing regions. A secondary, exploratory goal was to assess potential relationships between these neural patterns and affective characteristics within a normative healthy adolescent sample.

## 2. Methods

### 2.1. Participants

Forty-five healthy adolescents (13-17 years; mean age=15.8, SD=1.4; n=27/60% female) were recruited from the San Diego area via e-mail, internet, or flyers. Mean IQ, based on the Wechsler Abbreviated Scale of Intelligence/WASI (Wechsler, 1999), was 105.9 (SD=19.3). All participants provided written informed assent and their parent(s)/legal guardian(s) provided written informed consent in accordance with the Declaration of Helsinki and the Institutional Review Boards at the University of California, San Diego (UCSD), UC San Francisco (UCSF), Rady Children’s Hospital in San Diego, and the County of San Diego. All participants were financially compensated for their time. All participants were right-handed, medically healthy, Tanner Stages 3 or above, and were not taking any medication or supplements at the time of assessment. They were also excluded for any Diagnostic and Statistical Manual of Mental Disorders (DSM-IV-TR) Axis I diagnosis or family history of mood or psychotic disorders in first- or second-degree relatives, as they were recruited as part of a larger study, which also included depressed adolescents (data not reported here; for a full list of inclusion and exclusion, see (Connolly et al., 2013). Since we present a novel paradigm, we wished to initially present the results of the healthy adolescents in order to fully describe and detail our unpublished new task that can be utilized to understand the typical neurodevelopment of the adolescent brain in this key and underexamined area.

### 2.2. Experimental Procedures

To determine the presence of any DSM Axis I disorders, all participants were administered the computerized Diagnostic Interview Schedule for Children version 4.0 (Shaffer et al., 2000) and the Diagnostic Predictive Scale (Lucas et al., 2001). Family history of mood or psychotic disorder was assessed with the Family Interview for Genetic Studies (Maxwell, 1992). All participants underwent a clinical assessment, self-paced completion of questionnaires assessing mood and emotional characteristics, and completion of the experimental task while undergoing fMRI. Self-report measures included the Behavioral Inhibition System and Behavioral Activation System (BIS/BAS) Scales (Carver and White, 1994), Tanner Stage (Marshall and Tanner, 1969, 1970), and the Emotional Susceptibility and Irritability scales (Caprara et al., 1985).

### 2.3. Experimental Task

**Figure 1.**
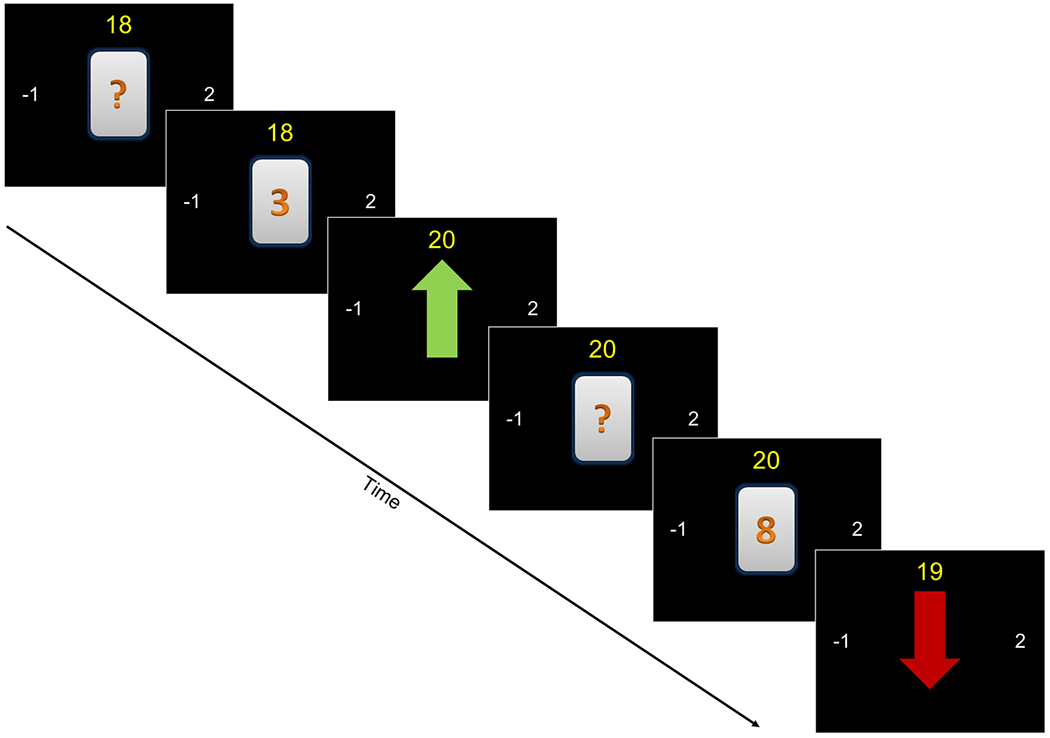
Frustration and Reward Task. Example of two successive trials in the high-stake condition, i.e., in which a win increases points by 2 and a loss decreases points by 1 (indicated by the values on the left and right side of the screen). Each trial is composed of three events: 1) a first screen presents a hidden card with a question mark to prompt participants to guess whether the value on the card (range=1 to 9) is below or above 5, by pressing ‘1’ or ‘2’ on the button box respectively; 2) upon button press, a second screen reveals the value on the card, presented for 500ms; 3) a third screen reveals the outcome of the trial for 500 ms, i.e., either a ‘win’ (green arrow pointing upward) or a ‘loss’ (red arrow pointing down) and points are adjusted accordingly (number on top of the screen). In this example, the <5 option was selected twice in the row, which was correct in the first trial and resulted in a win of +2pts, but was incorrect in the second trial, resulting in a loss of 1 pt. On blocks with blocked action efficacy (8/16 blocks), button presses were not working, i.e., the ‘?’ card would stay on the screen, for 1000-5000ms increments, until a response was eventually registered.

The present gambling task was developed based on previously validated tasks that successfully parsed out neural activity associated with reward anticipation as a function of stake in both adults (Delgado et al., 2000) and adolescents (Forbes et al., 2009). The task is self-paced, and participants were instructed to make their choices to maximize total points but did not know any other details about the task. Each trial is composed of three events: 1) a first screen presents a hidden card with a question mark to prompt participants to guess whether the value on the card is below or above 5, by pressing the 1^st^ and 2^nd^ button on an MRI scanner-compatible button-press box, respectively; 2) a second screen appears immediately upon button press and reveals the value on the card, presented for 500ms; 3) a third screen reveals the outcome of the trial for 500ms, i.e., either a ‘win’ (green arrow pointing upward) or a loss (red arrow pointing down), and points are adjusted accordingly (number on top of the screen; see Figure 1). Card values (range:1-9) were randomly determined with equal chances (44.4%) to win or lose (5 being considered a tie). Participants completed a total of 16 blocks of 28 seconds minimum, after which the next block was initiated. These included 8 blocks without any action efficacy obstruction (i.e., button presses working correctly and registering the participant’s response), in which participants made responses at a normal pace, and 8 blocks during which button presses were seemingly not working for increments randomly sampled between 1000ms and 5000ms, until a response is registered (aimed at inducing frustration in the participants). Thus, the number of completed trials per block varied depending on participants’ pace but participants spent equivalent amounts of time performing the task with and without response efficacy obstruction. Within each condition, half the blocks (*n*=4) had low-stake potential outcomes, i.e., +1 if correct, 0 if incorrect guess; the other blocks (*n*=4) had high-stake potential outcomes, i.e., +2 if correct, −1 if incorrect guess. For all participants, these blocks were presented in alternating order to control for order effects, starting with a normal, unobstructed block with high-stake trials. Finally, a total of 5 fixation cross screens of 16 seconds were presented at the beginning and between every other block, which served as baseline.

To analyze participants’ choices on unobstructed trials as a function of stake condition (Delgado et al., 2000), we considered the additional role of previous outcome in guiding the decision process given evidence that a win-stay/lose-shift heuristic reliably captures behavior in similar gambling tasks (Paulus et al., 2001; Worthy et al., 2013; Worthy and Maddox, 2014). A generalized linear mixed-effect model (GLME) was fit to binary decisions coded to represent whether one selected the same side of the deck as the one selected on the previous trial (1=stay) or whether the other side was selected (0=shift). The model assumed a binomial distribution and logit link function for binary outcome data and included both the current trial stake (i.e., low or high) and previous outcome (i.e., win or lose) as predictors. For obstructed blocks, the outcome of interest was the number of perseverative responses to action efficacy obstruction (i.e., button presses not registering an answer following the first obstructed response). Number of perseverative responses and associated response times per block were analyzed with GLMEs. The main effects of stake and block number on these outcome variables, as well as their interaction effects, were assessed. Given the positive skew of the reaction times, a Gamma distribution was used in associated GLMEs (Lo and Andrews, 2015).

### 2.4. fMRI Analyses

#### 2.4.1. Image acquisition

Participants were scanned on a 3T GE Discovery MR 750 System (Milwaukee, WI). For the whole task duration, T2* echo planar images (EPI) were acquired in contiguous slices (256 volumes TR = 2 s, TE = 30ms, flip angle = 90°, FOV 192 mm, 64 × 64 matrix, 3 × 3 mm in-plane resolution, 40 3mm axial slices) to measure blood oxygen level dependent (BOLD) signal). A high-resolution T1-weighted structural image was also collected (TR = 8.1ms, TE = 3.17ms, flip angle = 12°, 256 × 256 matrix, 168 1-mm sagittal slices, 1 × 1 mm in-plane resolution) to facilitate functional localization.

#### 2.4.2. Pre-processing and individual-level analysis

Preprocessing, normalization to MNI coordinates, and subsequent fMRI analyses were conducted using Analysis of Functional NeuroImages (AFNI) software (Cox, 1996). Preprocessing steps included removal of temporal outliers and volumes with excessive motion, as well as slice time correction and motion correction. Final resolution of functional images was 3×3×3 mm^3^. Individuals with >10% of brain volumes censored because of excessive motion were excluded from the analyses (two participants in total). One additional participant was removed from the analyses due to incomplete spatial collection of volumes. Thus, data from 42 participants were included in these fMRI analyses. Functional data were aligned to individuals’ anatomical and MNI templates. For each individual, a total of 16 regressors of interest were generated for specific event types. For all event types, the trial period from decision phase onset (i.e., end of previous trial outcome or previous button press if no outcome was presented on that previous trial) to the current trial button press was convolved with a canonical hemodynamic response function (HRF). More specifically, for decision events during unobstructed blocks occurring after the first obstructed block (median number of completed trials per block = 18, mean=19.7), 8 regressors were generated capturing the combination of 2 stake conditions (high vs low) X 2 types of outcome (win vs loss) and 2 types of subsequent decision (stay with versus shift to a different number guess category, e.g., <5 or >5). To maximize variance explained in our model, and given that the first block was uniquely experienced prior to encountering any action obstruction, 4 separate regressors were created for this first unobstructed, high-stake block with the same decision type (stay with vs shift) X previous outcome (win vs loss) break down. For events encountered during the obstructed phase (i.e., blocked button presses), 4 regressors were generated, including unobstructed trials (median number of completed trials per block=6, mean=6.2) and obstructed, perseverative button presses (median number of obstructed button presses=13, mean=17.1, i.e., about 73% of button presses in these blocks), each type of trial being distinctly regressed for low vs high-stake trials (see Supplementary Methods for regressor diagnostic statistics). Given the relatively lower number of completed trials with outcomes in the obstructed blocks, these trials were not further separated based on previous outcome, nor were they directly contrasted with completed trials in unobstructed blocks (see below). Finally, six motion regressors as well as linear and baseline trend regressors were added to the model.

#### 2.4.3. Group-level analysis

Analysis of unobstructed blocks occurring after the first obstructed block focused on post-reward decision trials to gauge the neural correlates of unobstructed reward processing in the context of intermittent obstruction of action efficacy. Voxel-based *t*-tests (with AFNI 3dttest++ function) were conducted for each of the 4 post-reward decision regressors described above (i.e., win-stay and win-shift, both in low- and high-stake). An additional contrast averaging all 4 types of reward trials (across stake and decision type) was also conducted (see Supplementary Table S1). Given the absence of a behavioral effect of loss on subsequent decision patterns as a function of stake (see section 3.1.1 below), we only assessed neural activity of post-loss decisions in each stake condition and as whole (averaged across stake conditions, see Supplementary Tables S2–S3).

To assess the impact of action efficacy obstruction on neural activity associated with reward-based decision-making, voxel-based *t*-tests were conducted for each of the two perseverative response regressor in low- and high-stake (i.e., during blocked action efficacy; see Supplementary Table S4 for overall effect of perseveration across stake conditions). A paired *t*-test contrast comparing low vs high perseverative response was further conducted to identify regions exhibiting differential activation patterns during those trials as a function of stake. Since obstructed blocks primarily included obstructed button presses and had significantly fewer completed trials than unobstructed blocks, we did not directly contrast completed decision trials between unobstructed and obstructed blocks in our analyses, but rather accounted for these distinct phases in our GLM (see section 2.4.2. above). To correct for multiple comparisons, we used a cluster-based thresholding method based on Monte-Carlo simulations implemented in 3dClustSim which accounts for smoothing using a permutation test based method (Cox et al., 2017). A voxel-wise *a priori* probability of 0.001 was used, which resulted in a corrected cluster-wise activation probability of 0.05 using a minimum volume of 11 connected voxels. Average percent signal change was extracted from significant clusters associated with blocked action efficacy in low- and high-stake conditions. These activations were correlated with several behavioral measures of interest, including participants’ age, level of behavioral activation and inhibition (as measured by 4 subscales of the BIS/BAS questionnaire, i.e., BAS Drive, BAS Fun Seeking, BAS Reward Responsiveness, BIS), and trait frustration (measured by the Irritability and Emotional Susceptibility scales).

## 3. Results

### 3.1. Behavioral Analyses

#### 3.1.1. Unobstructed blocks: decision choices as a function of stake.

**Figure 2.**
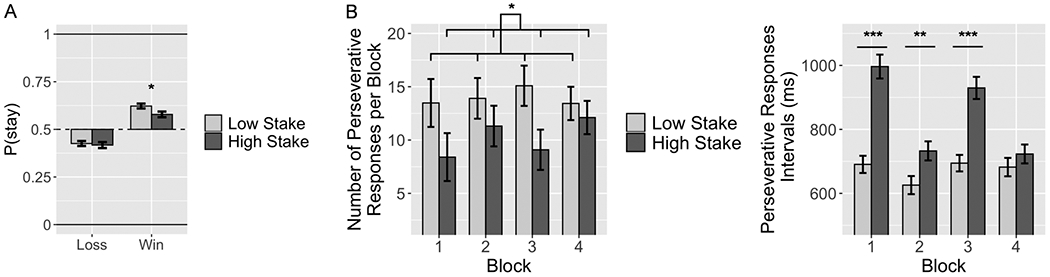
A. Behavioral Performance. Probability of choosing the same card guess option (i.e., > or < 5) following a win (“win-stay”) or a loss (“lose-stay”) as a function of stake (low, i.e., 0/1 vs high, i.e., −1/2); the dotted line indicates a 50% rate of staying with the same option (or switching), i.e., chance level for either strategy. **B.** Left: average number of perseverative responses per block during the obstructed blocks as a function of block and stake condition; Right: perseverative response time intervals as a function of block and stake; error bars=SEM; *,**,*** indicate a statistically significant difference at *p*<0.05, *p*<0.01, *p*<0.001, respectively.

Across blocks following the first obstructed block, participants were more likely to pick the same guess option on the following trial after a win, i.e., “win-stay”, relative to a loss (OR=2.19, z=13.0, *p*<0.001). Specifically, participants were more likely to win-stay (60.3%), which was significantly above 50% chance (OR=1.21, *p*<0.001). Participants were also more likely to switch to the other guess option on the subsequent trial in response to a loss, i.e., “lose-shift” (42.2%; OR=0.84, *p*=0.002). Finally, participants were significantly more likely to win-stay in low-stake (62.2%) relative to high-stake trials (57.8%; OR=1.21, z=2.2, *p*=0.028), but they had similar lose-stay rates in both low (42.6%) and high-stake trials (41.7%, z=0.28, *p*=0.778; see Figure 2A). Age and affective measures (i.e., BIS/BAS subscales, Irritability and Emotional Susceptibility scales) were not related to choice type (*p*s>0.05).

#### 3.1.2. Obstructed blocks: perseverative responses as a function of stake.

During obstructed blocks, participants made an average of 12 perseverative responses per block. A significant main effect of stake was observed, with more perseverative responses on low-stake (M=13.9) relative to high-stake trials (M=10.2; *p*=0.002; *d*=0.24; see Figure 2B). Neither a main effect of block (*p*=0.315) nor a block 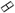 stake interaction (*p*=0.431) was observed. A significant effect of stake on perseverative response times revealed longer response time intervals on high-stake (M=845ms) relative to low-stake trials (M=673ms; *p*<0.001, *d*=0.29). There was no main effect of block (*p*=0.516), but there was a significant block 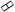 stake interaction (*p*<0.001), such that there were significantly longer responses time in blocks 1-3 (*ps*<0.05) but not in block 4 (*p*=0.920; see Figure 2B). Age and affective measures (i.e., BIS/BAS subscales, Irritability and Emotional Susceptibility scales) were not related to the number of perseverative responses or time between perseverative responses (*p*s>0.05).

### 3.2. Neural mechanisms associated with unobstructed reward processing

#### 3.2.1. Low-Stake

On low-stake trials (i.e., +1pt/0pt), when deciding to stay with the same option (i.e., win-stay trials), significant activation was found in DMN regions (i.e., bilateral precuneus), visual cortex, and areas associated with goal directed behavior (i.e., dorsal anterior cingulate cortex, i.e., Brodmann Area/BA 32, dorsolateral prefrontal cortex/BA 9) and with reward processing (i.e., putamen). Deactivations were observed in somatosensory processing areas (i.e., postcentral gyrus, posterior insula; ps<0.001; see Table 1 top panel). On low-stake trials on which participants decided to shift to the other choice option (i.e., win-shift trials), significant activations were observed in the inferior parietal lobules, dorsolateral prefrontal cortex (BA 9), and dorsal anterior cingulate (BA 32), whereas deactivations were observed in the postcentral gyrus and posterior cingulate cortex (ps<0.001; see Table 1 bottom panel).

#### 3.2.2. High-Stake

On high-stake trials (i.e., +2pt/−1pt), when deciding to stay with the same option in response to a win (i.e., win-stay trials), significant activation was found in the occipital cortex, precentral gyrus/premotor cortex (BA 6), dorsolateral prefrontal cortex, dorsal anterior cingulate gyrus (BA 32), and caudate. A few deactivation clusters were observed in the posterior cingulate cortex (BA 31), posterior insula, and temporal areas (see Table 2 top panel). When deciding to shift to the other guess option (i.e., win-shift trials), significant activations were observed in the dorsal anterior cingulate cortex (BA 32), dorsolateral prefrontal cortex (BA 9), precentral gyrus/premotor cortex (BA 6), caudate, and inferior parietal lobules (BA 40). Significant deactivations of DMN regions (i.e., posterior cingulate cortex, ventral anterior cingulate cortex) and somatosensory and somatosensory processing areas (i.e., bilateral posterior central gyri/BA 3) were also observed (see Table 2 bottom panel).

### 3.3. Neural response to obstructed reward processing

Regions of significant neural activation to button press obstruction were assessed for each stake condition, and for the specific contrast of high vs low-stake (see Supplementary Table S4 for activation clusters associated with blocked action efficacy across stake conditions).

#### 3.3.1. Low-Stake

A total of 11 regions were identified in which significant deactivations were observed during perseverative responses on low-stake trials (i.e., +1pt/0pt). These areas included DMN regions (e.g., a large bilateral cluster in the posterior cingulate/precuneus, i.e., BA 31/7), as well as areas associated with goal-directed behavior, i.e., dorsolateral prefrontal cortex (BA 9), precentral gyrus/premotor cortex (BA 6), dorsal anterior cingulate (BA 32); and reward processing (i.e., caudate; see Table 3 top panel). Average perseverative button presses per block, age, and affective measures (i.e., BIS/BAS subscales, Irritability and Emotional Susceptibility scales) were not related to activation in these regions (ps>0.05).

#### 3.3.2. High-Stake

**Figure 3.**
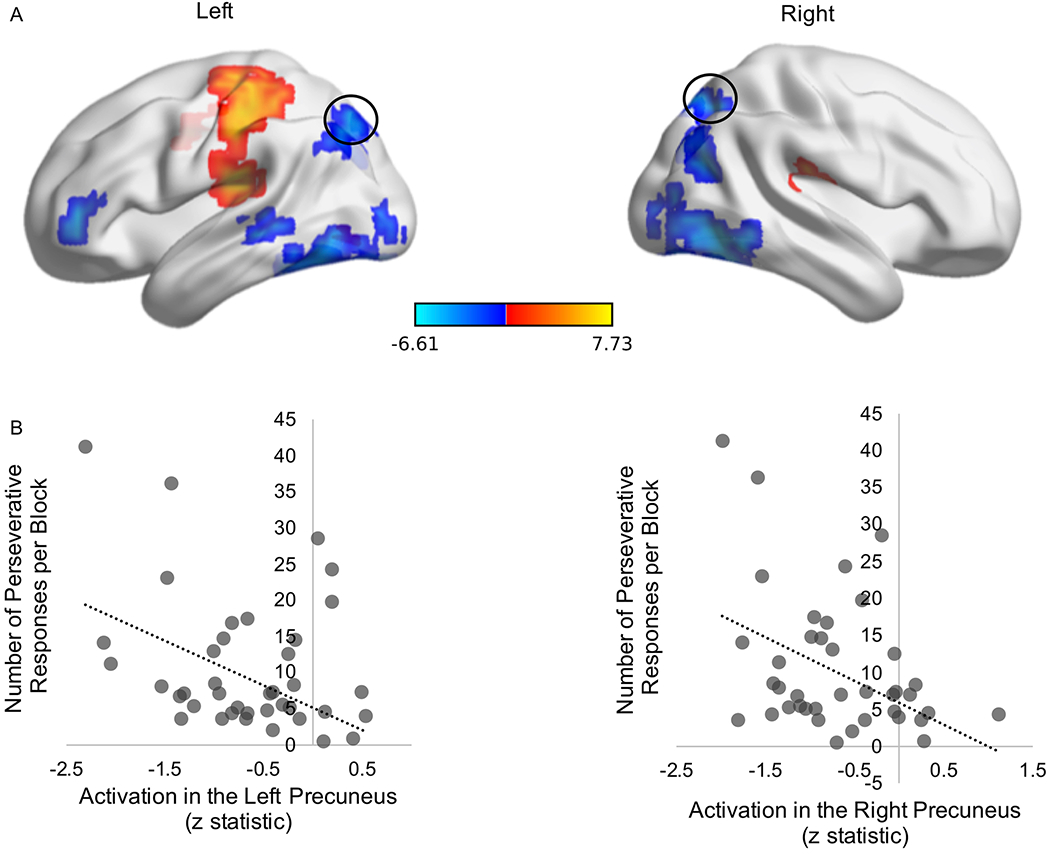
A. High-Stake Condition. Activation clusters associated with a significant change in BOLD signal during perseverative responses on all high-stake trials (i.e., +2pt/−1pt), including 2 clusters in the left and right precuneus (black circles; color bar reflects t statistic). **B.** Participants’ average amount of perseverative responses during high-stake trials was negatively correlated with average cluster activation (individual coefficient z statistic) in the left precuneus (*r*=−.32, *p*=0.041) and **C.** the right precuneus (*r*=−.38, *p*=0.014), respectively.

Another set of 11 regions showed a significant effect during perseverative responses on high-stake trials (i.e., +2pt/−1pt). Reduced activity was observed in visual processing regions (i.e., cuneus, occipital cortex), lateral prefrontal cortex (BA10), and DMN areas (i.e., left and right precuneus/BA 7). Clusters in the left and right posterior insula as well as the left post-central gyrus (BA 2) exhibited increased activation (see Table 3 middle panel). Average BOLD signal change in the left and right (see Figure 3A) precuneus, but no other identified clusters, were negatively correlated with average number of perseverative responses per block (ps<0.05; see Figure 3B). Age and affective measures (i.e., BIS/BAS subscales, Irritability and Emotional Susceptibility scales) were not related to activation in these clusters (ps>0.05).

#### 3.3.3. High- vs Low-Stake

**Figure 4.**
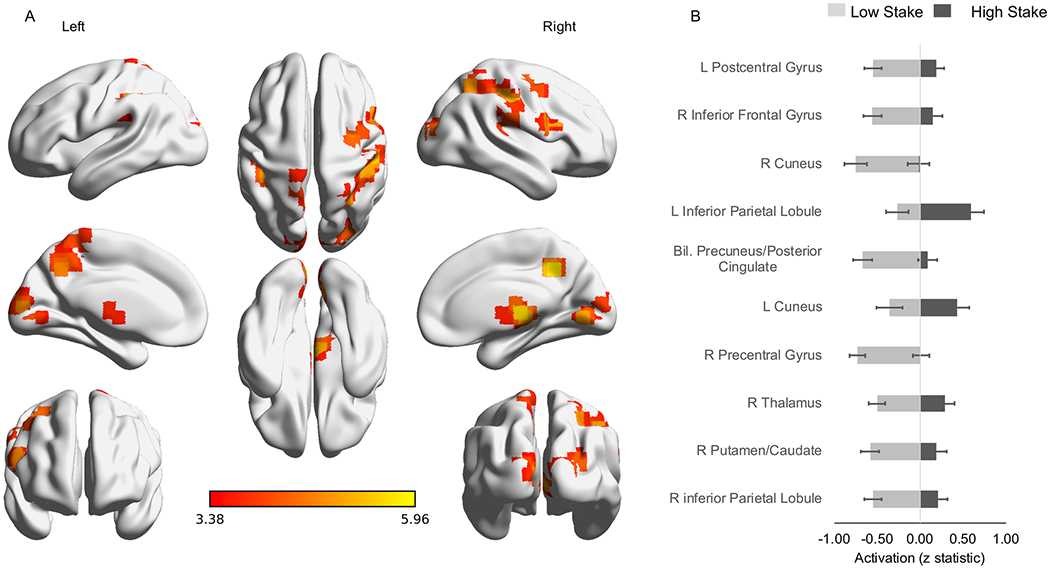
High- vs Low-Stake Condition. **A.** Activation patterns associated with a significant difference in neural activation during obstructed perseverative responses in high-stake vs low-stake trials in the bilateral precuneus (color bar reflects t statistic). **B.** Across all activation clusters, significantly more deactivation was observed in low-stake relative to high-stake (ps<0.001).

Assessing for the specific contrast of high vs low-stake obstructed trials, 10 regions were identified with a significant difference in neural activity between stake conditions. These included DMN regions (bilateral posterior cingulate/precuneus, i.e., BA 7), areas associated with goal-directed decision-making (i.e., left and right inferior parietal lobules, right precentral gyrus/premotor cortex, right lateral/inferior frontal gyrus, right caudate), and regions associated with affective and interoceptive processing (e.g., right thalamus, left postcentral gyrus); see Table 3 bottom panel, Figure 4A). In all clusters, more deactivation was observed in the low relative to high-stake condition (see Figure 4B).

## Discussion

In the present study, we describe a novel task designed to examine neural activity in response to action efficacy obstruction (i.e., a condition with low agency and high frustration potential) during reward-based decision-making among healthy adolescents. While undergoing fMRI, participants performed a card game in which they had to guess between two options to earn points, with different stakes. When performing the task without action efficacy obstruction, expected neural regions supporting goal-directed decision-making and reward processing were engaged. During action efficacy obstruction, we observed three main findings. First, the precuneus, a DMN region, was significantly less deactivated in high relative to low-stake trials, and fewer perseverative button presses were associated with less deactivation in both the right and left precuneus. Second, higher brain activity in affective and interoceptive processing areas was observed on high-stake relative to low-stake trials. Third, on low-stake decision trials, deactivations of areas associated with goal-directed action and reward processing – particularly the caudate and frontoparietal regions – were observed, and this network of regions was significantly less deactivated in the high-stake condition. Overall, our results are consistent with 1) the maintenance of task engagement, but diminished goal-directed reward seeking during blocked action efficacy; and 2) a stronger introspective focus and affective processing when outcome stakes are higher. We summarize and discuss our results in more detail below.

Our adolescent participants recruited expected neural regions associated with goal-directed reward seeking behavior when engaged in the task without action efficacy obstruction. During low-stake trials, staying with the same choice option following a win (i.e., win-stay) was more prevalent than a switching strategy. This behavior was associated with higher activation in the DMN, including bilateral precuneus and anterior cingulate cortex, as well as areas supporting goal-directed behavior (i.e., dorsolateral prefrontal cortex, premotor cortex) and reward processing (i.e., putamen). In contrast, deactivations were observed in somatosensory processing areas (i.e., posterior insula, somatosensory cortex). Overall, this is consistent with goal-directed behavior but reduced task engagement and emotional processing that is aligned with a more automatic, win-stay behavior. On higher stake trials (with twice the winning potential, but also with greater risk of loss instead of reward omission), adolescents were more likely to explore different choice options following a win (i.e., win-shift) rather than staying with the same option. In this condition, higher neural activity was observed in a more extensive set of central executive areas involved in goal-directed decision-making (i.e., premotor cortex, inferior parietal lobule, caudate). However, significant deactivations were observed in DMN regions (i.e., posterior cingulate cortex, anterior cingulate cortex) and somatosensory and affective processing areas (i.e., insula, postcentral gyrus). Overall, these findings are consistent with more task engagement and external focus during goal-directed reward seeking behavior on high-stake trials, suggesting the higher propensity to win-shift observed in this condition is more likely to reflect increased exploration of the reward environment rather than random choice.

While deactivations of the precuneus, a DMN region, was observed during blocked action efficacy in both low- and high-stake conditions, suggesting increased task engagement and reduced internal focus, the high-stake condition was associated with less deactivation of the left and right precuneus along with fewer and slower perseverative button presses. Deactivation in the DMN, particularly the posterior cingulate/precuneus region, has been observed in irritable youths during frustration manipulations (Deveney et al., 2013; Perlman et al., 2015; Tseng et al., 2019). In the absence of deactivations in goal-directed decision-making and reward processing areas, this suggests that the high-stake condition was more motivating, as suggested by a flexible behavioral adjustment (i.e., decrease in perseverative button presses). For instance, the smaller precuneus deactivation could reflect more internally focused attention and strategic adjustment to slow down and being more mindful in a situation with higher stakes. In contrast, the higher level of perseverative responses and stronger precuneus deactivation in the low-stake condition may reflect the idea that more reactive and impulsive behaviors are engaged during lower stakes (and, indeed, this interpretation is consistent with the fact that there was a higher rate of win-stay choices observed in the unobstructed low-stake condition).

Alternatively, failure to deactivate DMN regions in the high-stake condition may reflect the intrusion of internal cognition associated with increased frustration relative to a less consequential (i.e., low-stake) context. Higher activity in DMN regions is observed during the experience of discrete emotional states, such as anger (Satpute and Lindquist, 2019). Altered deactivation in the posterior cingulate/precuneus during blocked action efficacy on high-stake trials is congruent with prior findings of reduced deactivations in those regions during emotional processing and rumination in adolescents with current or history of depression (Ho et al., 2015; Burkhouse et al., 2017). However, since frustration and anger can be characterized as being externally driven, approach-based emotions (Harmon-Jones and Allen, 1998; Carver and Harmon-Jones, 2009), stronger frustration in the high-stake condition would be expected to lead to more rather than fewer perseverative button presses relative to the low-stake condition. Future research should seek to further delineate the relationship between DMN region deactivation during frustration and mood dysregulation in adolescents.

Blocked action efficacy during high-stake trials was also uniquely associated with increased activity in the postcentral gyrus and posterior insula. In addition, less deactivation in the thalamus and postcentral gyrus was observed in the high relative to low-stake condition. Both the posterior insula and postcentral gyrus have been robustly implicated in affective and interoceptive processing (Adolphs et al., 2000; Craig, 2002; Harrison et al., 2010; Saxbe et al., 2013), including the experience of negative emotional states such as disgust (Calder et al., 2007) and anger (Sorella et al., 2021). As part of the cingulo-opercular/salience network, the thalamus is an important somatosensory processing hub with extensive reciprocal connections to the prefrontal cortex (Seeley et al., 2007; Sadaghiani and D’Esposito, 2015), which is implicated in the regulation of arousal and alertness (Van der Werf et al., 2002; Sadaghiani and D’Esposito, 2015). In the context of this research, our findings of increased activity in the left insula and postcentral gyrus during high-stake trials may indicate that adolescents have a more embodied emotional experience in this condition, presumably stemming from higher level of frustration (Saxbe et al., 2013). As mentioned above, though, this explanation is not consistent with the lower rate of perseverative button presses and absence of deactivations in goal-directed reward seeking regions in this condition.

Higher activity in the somatosensory cortex and thalamic areas could instead reflect a behavioral adaption to optimize performance in light of increased attentional needs, as previously observed following stressors and frustration induction (Bierzynska et al., 2016). As part of the somatosensory cortex, the postcentral gyrus plays an important role in tactile and visuospatial attention, with attention modulating activity in this area (Mima et al., 1998; Waberski et al., 2002; Balslev et al., 2013). The fact that high-stake trials were associated with fewer perseverative responses further suggests that this increased activation of somatosensory cortex is unlikely to reflect increased sensory stimulation related to such behavior. Taken together, these findings are consistent with the formulation that obstruction of action efficacy in a high-stake condition may promote a stronger interoceptive, and potentially affective, processing response. However, we did not collect psychophysiological measures of affective processing and attention in the present study. Such methods in future studies could be useful in disentangling the role of the insula and somatosensory cortex in response to frustration in adolescents.

Significantly more deactivations in the low-stake relative to high-stake trials were observed in the right putamen/caudate, right precentral gyrus, right inferior/lateral frontal gyrus, and bilateral inferior parietal lobules. The caudate and putamen are involved in learning, motivation, and reward processing (O’Doherty et al., 2004; Delgado et al., 2005; Haruno and Kawato, 2006). The precentral gyrus/premotor cortex (BA 6) has been implicated in behavior planning and execution, particularly learning stimulus-motor relationships (Petrides, 2005; Badre and D’esposito, 2009), while the lateral prefrontal cortex and posterior parietal areas, including inferior parietal lobule, are part of the frontoparietal network involved with task control (Seeley et al., 2007; Ptak, 2012). Importantly, the deactivations observed during the low-stake obstructed trials were in prefrontal, parietal, and striatal areas similar to those recruited during unobstructed decision blocks, consistent with their role in supporting reward exploration and decision-making in the present paradigm. Taken together, these findings are consistent with the formulation that obstructing action efficacy when stakes are low is associated with decreased goal-directed decision-making pertaining to maximizing rewards in the task, whereas such goal-directed processes are significantly more preserved in the high-stake condition. This idea is congruent with a flexible and adaptive mechanism to deallocate cognitive resources from goal-driven behavior in the face of obstruction when stakes are relatively low, but less so when stakes and related motivation are higher (Kolling et al., 2016; Shenhav et al., 2016). These findings are also consistent with models of perceived agency and controllability, constructs relevant to one’s ability to learn action-outcome contingencies in the environments and to adjust behavior accordingly (Moscarello and Hartley, 2017; Ly et al., 2019). In the present paradigm, blocking action efficacy decreases perceived agency and control as it prevents individuals to observe and learn choice-outcome contingencies. This ‘low control’ context would be expected to promote a more reactive behavioral strategy (Moscarello and Hartley, 2017) and persistence of reflexive, Pavlovian behavior (Dorfman and Gershman, 2019). Here, we find that high-stake anticipation may contribute to a reduction in this tendency and preserve a higher degree of goal-directed behavior in adolescents. Such moderating effects, whereby outcome-based elements (e.g., higher average reward rate and/or reward probability) may increase perceived controllability and goal-directed behavior, have been previously observed (Niv et al., 2007; Tobias-Webb et al., 2017; Ly et al., 2019). Importantly, recent modeling work suggests that estimation of controllability for reward outcomes and its use in guiding action valuation and decision-making are observed in both adults and adolescents relative to younger children (Cohen et al., 2020), consistent with our findings in an adolescent sample.

The present study’s findings should be interpreted in light of its limitations. A relatively modest sample size and age range limits the generalization of these findings to a wider-aged healthy adolescent population. However, limiting the age range and restricting the participants to those who were postpubertal (Tanner Stages 3 or above) also reduced potential heterogeneity in the findings. While age was not related to either behavioral or neural markers of blocked action efficacy, this does not preclude the presence of developmental effects in a broader age range from childhood to young adulthood. We also did not measure self-report or psychophysiological markers of state frustration during the task, which limits the interpretation of the present findings with regards to emotional experience during the response obstruction blocks. Given the potential confounds associated with collecting self-report affective measures during decision-making (Pham, 1998; Schwarz, 2004), concurrent psychophysiological measures of affective valence and arousal, such as facial electromyography and skin conductance response, would be important to consider to further validate the affective impact of this novel paradigm in youth. Yet, based on the lower level of perseverative responses and specific neural patterns observed during high-stake obstructed trials, our findings suggest that blocking action efficacy alters neural and behavioral responses as a function of value outcomes, such that higher stakes may elicit more thoughtful (i.e., less perseverative) responses and stronger internal and affective focus. This contrasts with a more reactive and impulsive response to blocked action efficacy, with greater motor perseveration, when stakes are lower. Thus, adolescent participants were engaged in the task and were sensitive to contextual effects (i.e., stake condition). Finally, while providing insights into how an incidental source of frustration characterized by reduced agency may impact reward processing, the present study does not address how this type of frustrative non-reward compares to reward omission or loss, events that may more directly shape individuals’ beliefs about the reward environment (e.g., expected reward rates). Future research should seek to disentangle those different types of frustrative non-reward.

In conclusion, we describe for the first time a novel fMRI task that was designed to study neural activity associated with the experience of blocked action efficacy during reward-based decision-making as a behavioral assay of frustration among healthy adolescents. We found that blocking effective responding during reward-based decision-making on high-stake relative to low-stake trials was associated with smaller deactivations in DMN areas (i.e., precuneus) and in regions supporting goal directed reward-based decision-making (i.e., putamen/caudate and frontoparietal task control regions). Our results in healthy adolescents are consistent with the maintenance of task engagement and reduced goal-directed behavior when being blocked from making reward-based choices, albeit to a smaller extent and with stronger internally driven attention and affective processing when reward stakes are higher. Our findings have implications for the real-time impact of incidental frustrating events on reward processing in adolescence, a key neurodevelopmental period marked by significantly higher risk for increased irritability, depression, and impulsive behavior.

## Supplementary Material

1774132_Sup-material

## Figures and Tables

**Table 1.** Activation associated with WIN outcomes during LOW-STAKE blocks.

Side	Region	BA	Volume (voxels)	x	y	z	t
**Win-Stay Low-Stake </> Baseline**							
R	Inferior Frontal Gyrus	10	13	37	46	6	4.379
R	Dorsal Anterior Cingulate Cortex	32	100	5	25	39	6.990
L	Dorsolateral Prefrontal Cortex	9	136	−41	12	24	5.681
L	Putamen/Caudate		102	−20	10	3	6.032
R	Inferior Frontal Gyrus	9/44	101	44	7	24	7.065
R	Putamen/Caudate		186	18	3	7	6.819
R	Posterior Insula	13	22	38	−20	10	−5.582
L	Posterior Insula	13	14	−38	−24	10	−4.674
R	Postcentral Gyrus	3	118	24	−36	50	−5.713
L	Postcentral Gyrus	3	20	−23	−37	57	−4.920
R	Precuneus	39/7	179	32	−58	38	7.703
L	Precuneus	39/7	152	−32	−58	37	6.937
R	Inferior Occipital Gyrus	37	277	40	−65	−12	7.856
L	Inferior Occipital Gyrus	19	211	−36	−70	−13	7.830
R	Cuneus	30	408	1	−77	3	−7.785

**Win-Shift Low-Stake </> Baseline**							
L	Dorsal Anterior Cingulate Cortex	32	497	−24	12	32	6.833
R	Dorsolateral Prefrontal Cortex	9/44	579	29	3	23	9.885
R	Postcentral Gyrus	3	19	32	−35	60	−4.513
L	Posterior Cingulate Cortex	31	36	−2	−40	40	−4.898
L	Inferior Parietal Lobule	40	159	−33	−51	37	7.203
R	Inferior Parietal Lobule	40	284	34	−55	36	8.129
R	Middle Occipital Gyrus	19	263	37	−69	−14	8.153
L	Lingual Gyrus	19	262	−34	−70	−15	7.435
L	Cuneus	17	74	−4	−80	7	−6.146

Note: L: left; R: right; BA: Brodmann area; x,y,z: Peak Voxel MNI coordinates; t: peak voxel t statistics, df= 41; p < 0.05 corrected (voxelwise p < 0.001); voxel size = 3x3x3 mm^3^; clusters are sorted by Y dimension (from anterior to posterior).

**Table 2.** Activation associated with WIN outcomes during HIGH-STAKE blocks.

Side	Region	BA	Volume (voxels)	x	y	z	t
**Win-Stay High-Stake </> Baseline**							
L	Caudate		45	−21	15	6	4.834
R	Dorsolateral Prefrontal Cortex	9/44	77	41	11	13	5.168
L/R	Dorsal Anterior Cingulate Cortex	32	52	2	10	46	7.674
R	Precentral Gyrus	6	16	21	0	56	4.973
L	Inferior Frontal Gyrus	9/44	15	−42	−2	24	6.138
L	Precentral Gyrus	6	50	−37	−8	49	5.854
R	Posterior Insula	13	18	41	−17	10	−5.091
L	Parahippocampal Gyrus	36	24	−23	−39	−14	−5.327
L	Posterior Cingulate Cortex	31	29	−6	−43	35	−5.472
L	Precuneus	7	20	−26	−57	36	5.371
L	Inferior Occipital Gyrus	19	101	−35	−69	−13	6.632
L/R	Lingual Gyrus		222	0	−74	−1	−6.580
R	Inferior Occipital Gyrus	19	69	38	−74	−6	7.033

**Win-Shift High-Stake </> Baseline**							
L/R	Ventral Anterior Cingulate Cortex	10/32	35	2	47	−2	−5.817
R	Dorsal Anterior Cingulate Cortex	24/32	234	22	5	38	8.339
L	Caudate/Anterior Insula		118	−4	3	8	7.661
L	Dorsolateral Prefrontal Cortex	6/9	49	−41	−4	34	5.464
L	Middle Frontal Gyrus	6	17	−27	−8	49	4.201
R	Posterior Insula	22/13	93	51	−13	5	−5.533
L	Posterior Insula	13	16	−36	−13	1	−5.904
R	Postcentral Gyrus	3	29	41	−22	49	−4.747
L	Postcentral Gyrus	40	13	−23	−43	58	−5.526
R	Postcentral Gyrus	2	37	23	−44	60	−6.012
L/R	Posterior Cingulate Cortex	31	61	−6	−45	40	−5.551
R	Inferior Parietal Lobule	40	75	34	−51	37	5.972
L	Inferior Parietal Lobule	40	75	−32	−51	38	6.098
R	Fusiform Gyrus	37	39	42	−64	−14	5.024
L	Inferior Occipital Gyrus	19	38	−37	−74	−10	5.479
L/R	Cuneus	18	558	0	−76	6	−9.244

Note: L: left; R: right; BA: Brodmann area; x,y,z: Peak Voxel MNI coordinates; t: peak voxel t statistics, df=41; p < 0.05 corrected (voxelwise p < 0.001); voxel size = 3x3x3 mm^3^; clusters are sorted by Y dimension (from anterior to posterior).

**Table 3.** Activation associated with BLOCKED ACTION EFFICACY during low- vs high-stake trials.

Side	Region	BA	Volume (voxels)	x	y	z	t
**Low-Stake Only </> Baseline**							
R	Ventral Medial Prefrontal Gyrus	10/32	73	17	50	−2	−6.748
R	Dorsal Anterior Cingulate Cortex	32	17	9	36	19	−4.684
L	Superior Frontal Gyrus	6	25	12	27	39	−5.085
L	Inferior Frontal Gyrus	46	41	−42	22	23	−4.907
L	Putamen/Caudate		90	−14	9	1	−6.104
R	Dorsolateral Prefrontal Gyrus	9	273	37	5	40	−7.815
R	Putamen/Caudate		230	21	−1	0	−7.905
L	Precentral Gyrus	6	102	−32	−4	46	−6.148
L	Premotor/Precentral Gyrus	6	12	0	−11	62	−4.620
R	Parahippocampal Gyrus	27	12	−16	−33	−4	−6.255
L/R	Precuneus	31	1903	6	−64	21	−9.299

**High-Stake Only </> Baseline**							
L	Anterior Prefrontal Cortex	10	27	−41	42	2	−5.196
L	Cingulate Gyrus	24	13	−4	−12	44	4.075
R	Posterior Insula	13/40	11	51	−25	18	4.371
L	Postcentral Gyrus/Insula	2	266	−42	−28	41	7.732
R	Culmen/Cerebellum	19	14	8	−62	−13	4.864
L	Inferior Occipital Gyrus	19	188	−38	−66	−9	−6.613
R	Middle Occipital Gyrus	37	131	42	−68	−7	−5.448
R	Precuneus	7	27	21	−68	49	−5.127
L	Precuneus	19/7	53	−32	−70	36	−4.880
R	Middle Temporal Gyrus	39	33	37	−70	30	−4.866
L	Cerebellum		12	−9	−71	−11	6.359

**High-Stake </> Low-Stake**							
R	Lateral/Inferior Frontal Gyrus	44	15	54	9	22	4.319
R	Putamen/Caudate		43	18	−1	12	5.073
R	Lateral Frontal/Precentral Gyrus	6	36	31	−8	47	5.563
R	Thalamus		41	11	−22	5	5.479
L	Inferior Parietal Lobule	40	18	−39	−36	37	5.719
R	Inferior/Superior Parietal Lobule	40	85	38	−38	41	5.964
L	Postcentral Gyrus	3	15	−13	−42	63	4.098
L/R	Precuneus	7	31	−3	−53	43	5.012
R	Cuneus	18	15	24	−83	17	4.926
L	Cuneus	17	34	−3	−84	7	5.304

Note: L: left; R: right; BA: Brodmann area; x,y,z: Peak Voxel MNI coordinates; t: peak voxel t statistics, df=41; p < 0.05 corrected (voxelwise p < 0.001); voxel size = 3x3x3 mm^3^; clusters are sorted by Y dimension (from anterior to posterior).
