# Supplementary material for: The effect of obstructed action efficacy on reward-based decision-making in healthy adolescents: a novel functional MRI task to assay frustration
Source: Cogn Affect Behav Neurosci. Author manuscript; Available in PMC 2023 Jun 1. (PMC9090962; doi:10.3758/s13415-021-00975-w)
Supplement: 1774132_Sup-material [file NIHMS1774132-supplement-1774132_Sup-material.pdf]

SUPPLEMENTARY MATERIAL

Supplementary Methods

Details of 1st level GLM regressors:

The average number of trials and associated standard deviations (S.D.) across participants for each regressor in our model are presented in the table below. Numbers of trials ranged from 10.3 to 20.1 for the unobstructed blocks regressors of interest (after 1st block), and from 61.2 to 73.6 for perseverative response regressors (obstructed blocks; see shaded rows for these 10 regressors of interest). To ensure our model was not susceptible to collinearity, we obtained variance inflation factors (VIFs) for each model regressor, which ranged from 1.13 to 1.27 for regressors of interest (see last column in table below) and were below the conservative cutoff of 2 for problematic collinearity. We note that including the 4 regressors associated with the 1st block (regressors of no-interest) did not notably impact collinearity (VIFs range of 1.13 to 1. 81) relative to whether those regressors were not included (VIFs range of 1.12 to 1.26). However, accounting for these 4 1st-block regressors in our model allowed us to control for potential practice effects and other unique aspects of this block (first experience with the task prior to any action efficacy blocking).

Event Regressor	Mean	S.D.	VIF
Unobstructed Blocks (after 1st block)			
Low Stake - Win Stay	20.1	7.9	1.21
Low Stake - Win Shift	13.5	6.7	1.13
Low Stake - Lose Stay	13.8	6.8	1.16
Low Stake - Lose Shift	18.6	7.5	1.20
High Stake - Win Stay	14.8	6.1	1.27
High Stake - Win Shift	11.6	5.1	1.17
High Stake - Lose Stay	10.3	4.6	1.18
High Stake - Lose Shift	13.5	6.2	1.23
Obstructed Blocks			
Low Stake - Unblocked Outcome Trial	24.6	4.7	1.14
High Stake - Unblocked Outcome Trial	25.1	4.9	1.16
Low Stake - Blocked Perseverative Response	73.6	65.3	1.13
High Stake - Blocked Perseverative Response	61.2	38.5	1.14
Unobstructed Blocks (1st block, high stake)			
Win Stay	5.9	1.8	1.81
Win Shift	6.0	2.1	1.71
Lose Stay	5.3	1.9	1.70
Lose Shift	5.7	2.1	1.80

Supplementary Results

Table S1. Activation associated with WIN outcomes (across stake).

Hem	Region	BA	Volume (voxels)	x	y	z	t
L	Rostral ACC	32	18	-4	49	1	-6.083
L	Posterior Insula	13	111	-46	-16	5	-6.760
L/R	Thalamus/Caudate/Dorsal ACC		2902	1	-24	13	10.424
L/R	Posterior Cingulate Cortex	5/31	424	11	-37	48	-8.211
L	Inferior Parietal Lobule	40	233	-32	-54	38	7.891
R	Inferior Parietal Lobule	40	300	32	-57	36	8.884
R	Middle Occipital Gyrus	19	373	39	-66	-12	10.369

Note: Hem: hemisphere; L: left; R: right; BA: Brodmann area; x,y,z: Peak Voxel MNI coordinates; t: peak voxel t statistics, $p < 0.05$ corrected (voxelwise $p < 0.001$) with Monte Carlo simulations (via Analysis of Functional NeuroImages 3dClustSim) to guard against false-positive results; ACC=Anterior Cingulate Cortex; voxel size = 3x3x3 mm^3; clusters are sorted by Y dimension (from anterior to posterior).

Table S2. Activation associated with LOSS outcomes (across stake).

Hem	Region	BA	Volume (voxels)	x	y	z	t
R	Rostral ACC	32	338	2	46	4	-9.079
L	Orbitofrontal Gyrus	11/47	60	-35	27	-10	-5.431
L	Anterior Insula	13/47	28	-31	16	6	6.469
R	Ventral ACC/Caudate	25	28	0	13	-3	-5.858
L	Dorsolateral Prefrontal Cortex	9	133	-39	3	35	7.256
R	Precentral Gyrus/Dorsal ACC	6	605	32	2	26	7.502
R	Middle Temporal Gyrus	21	16	57	0	-17	-5.514
R	Caudate		12	17	0	11	4.998
L	Parahippocampal Gyrus	34	34	-22	-2	-16	-5.160
L	Superior Temporal Gyrus	22	14	-55	-3	3	-5.475
L	Middle Temporal Gyrus	21	36	-53	-7	-15	-5.695
L	Posterior Insula	13	59	-42	-20	10	-5.778
R	Inferior Parietal Lobule	40	506	32	-44	46	9.461
L/R	Posterior Cingulate Cortex	23	1569	-6	-62	17	-10.225
R	Fusiform Gyrus	37	188	39	-64	-13	7.581
L	Lingual Gyrus	18	124	-33	-72	-12	6.402
L	Middle Temporal Gyrus	39	45	-42	-74	19	-5.960

Note: Hem: hemisphere; L: left; R: right; BA: Brodmann area; x,y,z: Peak Voxel MNI coordinates; t: peak voxel t statistics, $p < 0.05$ corrected (voxelwise $p < 0.001$) with Monte Carlo simulations (via Analysis of Functional NeuroImages 3dClustSim) to guard against false-positive results; ACC=Anterior Cingulate Cortex; voxel size = 3x3x3 mm^3; clusters are sorted by Y dimension (from anterior to posterior).

Table S3. Activation associated with LOSS outcomes in low-stake and high-stake conditions.

Hem	Region	BA	Volume (voxels)	x	y	z	t
**Low Stake Loss (i.e., Reward Omission) > Baseline**							
R	Middle Frontal Gyrus	46	14	40	44	9	4.739
L/R	Ventral ACC	32	158	0	43	3	-6.531
L	Anterior Insula	13	14	-28	16	7	5.713
L/R	Dorsal ACC	32	127	2	11	45	7.336
R	Dorsolateral Prefrontal Cortex	9	297	40	9	32	7.262
L	Inferior Frontal Gyrus	9	184	-39	3	35	6.267
R	Posterior Insula	13	47	41	-20	13	-6.862
R	Cingulate Gyrus	24	22	1	-21	37	-5.000
R	Postcentral Gyrus	3/4	79	31	-32	52	-6.420
L	Paracentral Lobule/Precuneus	7	29	-26	-44	57	-6.039
L	Inferior Parietal Lobule	40	209	-33	-56	38	7.504
R	Inferior Parietal Lobule	40	324	33	-58	39	9.943
R	Fusiform Gyrus	37	217	40	-64	-13	7.983
L/R	Posterior Cingulate	30	649	-3	-70	8	-8.800
L	Lingual Gyrus	18	130	-32	-74	-12	7.874
**High Stake Loss > Baseline**							
L/R	Ventral ACC	32	193	2	49	3	-7.167
L	Orbitofrontal Cortex	47/11	48	-37	32	-9	-6.348
R	Anterior Insula	13	21	36	19	5	5.284
L/R	Dorsal ACC	32	54	3	12	41	5.920
R	Precentral Gyrus	6	47	41	-1	30	5.570
L	Posterior Insula	13	138	-44	-12	2	-6.220
R	Posterior Insula	22	154	48	-16	9	-6.371
R	Inferior Parietal Lobule	40	49	35	-51	38	5.805
R	Fusiform Gyrus	37	26	39	-52	-17	5.997
L	Inferior Parietal Lobule	40	15	-31	-52	36	5.617
L/R	Posterior Cingulate Cortex	23/30	1458	1	-59	22	-8.883

Note: Hem: hemisphere; L: left; R: right; BA: Brodmann area; x,y,z: Peak Voxel MNI coordinates; t: peak voxel t statistics, $p < 0.05$ corrected (voxelwise $p < 0.001$) with Monte Carlo simulations (via Analysis of Functional NeuroImages 3dClustSim) to guard against false-positive results; ACC=Anterior Cingulate Cortex; voxel size = 3x3x3 mm^3; clusters are sorted by Y dimension (from anterior to posterior).

Table S4. Activation associated with RESPONSE OBSTRUCTION (across stake).

**Hem**	**Region**	**BA**	**Volume (voxels)**	**x**	**y**	**z**	**t**
L	Medial Prefrontal Gyrus	10	22	-10	55	-1	-4.761
L	Inferior Frontal Gyrus	47	38	-39	43	-1	-6.399
L/R	Rostral ACC/Caudate	24	250	7	27	-3	-6.661
R	Inferior Frontal Gyrus	46	25	46	23	25	-5.372
L	Dorsolateral Prefrontal Cortex	9	135	-38	8	36	-6.444
R	Dorsolateral Prefrontal Gyrus	9	16	47	4	29	-4.796
R	Precentral/Middle Frontal Gyrus	6	77	31	-1	52	-6.011
L	Posterior Insula	13	42	-47	-25	18	6.443
L	Postcentral Gyrus	3	132	-39	-28	51	6.238
R	Cerebellum		14	7	-58	-14	5.016
L/R	Precuneus	23	1683	4	-66	14	-8.325

Note: Hem: hemisphere; L: left; R: right; BA: Brodmann area; x,y,z: Peak Voxel MNI coordinates; t: peak voxel t statistics, $p < 0.05$ corrected (voxelwise $p < 0.001$) with Monte Carlo simulations (via Analysis of Functional NeuroImages 3dClustSim) to guard against false-positive results; ACC=Anterior Cingulate Cortex; voxel size = 3x3x3 mm^3; clusters are sorted by Y dimension (from anterior to posterior).
